# The Type 3 Deiodinase Is a Critical Modulator of Thyroid Hormone Sensitivity in the Fetal Brain

**DOI:** 10.3389/fnins.2021.703730

**Published:** 2021-06-29

**Authors:** Maria Elena Martinez, Arturo Hernandez

**Affiliations:** ^1^Center for Molecular Medicine, Maine Medical Center Research Institute, MaineHealth, Scarborough, ME, United States; ^2^Graduate School for Biomedical Science and Engineering, University of Maine, Orono, ME, United States; ^3^Department of Medicine, Tufts University School of Medicine, Boston, MA, United States

**Keywords:** type 3 deiodinase, *Dio3*, thyroid hormone, brain development, *Klf9*, *Nrgn*

## Abstract

Thyroid hormones (TH) are critical for the development and function of the central nervous system (CNS). Although their effects on the rodent brain peak within 2–3 weeks postnatally, the fetal brain has been found largely insensitive to exogenously administrated TH. To address this issue, here we examined gene expression in brains from mouse fetuses deficient in the type 3 deiodinase (DIO3), the selenoenzyme responsible for clearing TH. At embryonic day E18.5 qPCR determinations indicated a marked increase in the mRNA expression of T3-responsive genes *Klf9* and *Nrgn*. The increased expression of these genes was confirmed by *in situ* hydridization in multiple areas of the cortex and in the striatum. RNA sequencing revealed 246 genes differentially expressed (70% up-regulated) in the brain of E18.5 *Dio3*−/− male fetuses. Differential expression of 13 of these genes was confirmed in an extended set of samples that included females. Pathway analyses of differentially expressed genes indicated enrichment in glycolysis and signaling related to axonal guidance, synaptogenesis and hypoxia inducible factor alpha. Additional RNA sequencing identified 588 genes differentially expressed (35% up-regulated) in the brain of E13.5 *Dio3*−/− male fetuses. Differential expression of 13 of these genes, including *Klf9, Hr*, and *Mgp*, was confirmed in an extended set of samples including females. Although pathway analyses of differentially expressed genes at E13.5 also revealed significant enrichment in axonal guidance and synaptogenesis signaling, top enrichment was found for functions related to the cell cycle, aryl hydrocarbon receptor signaling, PCP and kinetochore metaphase signaling pathways and mitotic roles of polo-like kinase. Differential expression at E13.5 was confirmed by qPCR for additional genes related to collagen and extracellular matrix and for selected transcription factors. Overall, our results demonstrate that the rodent fetal brain is sensitive to TH as early as E13.5 of gestational age, and suggest that TH distinctly affects brain developmental programs in early and late gestation. We conclude that DIO3 function is critical to ensure an adequate timing for TH action in the developing brain and is probably the main factor underlying the lack of effects on the fetal brain observed in previous studies after TH administration.

## Introduction

Thyroid hormones (TH) regulate the expression of a large number of genes in the developing brain, impacting the proliferation, migration and differentiation of multiple brain cell types, and ultimately exerting profound functional effects on the adult CNS (Legrand, 1984; Bernal and Nunez, 1995). Their action is largely mediated by 3,5,3′-triiodothyronine (T3), which can regulate gene transcription upon binding to its nuclear receptor, a DNA-binding transcription factor (Forrest and Visser, 2013). Processes critical for brain maturation such as neurogenesis, neuronal migration and maturation (Richard et al., 2020), dendrite formation, myelination and synaptogenesis are strongly regulated by TH (Bernal and Nunez, 1995; Bernal, 2005). In humans, reduced brain availability of TH during development leads to neurological abnormalities and, in extreme cases, to cretinism and Allan-Herndon-Dudley syndromes, which are characterized by severe intellectual disability and motor deficits (Legrand, 1984; Dumitrescu et al., 2004; Friesema et al., 2004). The importance of TH for the central nervous system is further underscored by the CNS abnormalities noted by studies on animal models with genetic alterations in genes regulating brain TH availability and action. Thus, broad neurological, sensory and behavioral phenotypes are noted in mice with deficits in TH receptors (Dellovade et al., 2000; Venero et al., 2005; Siesser et al., 2006; Wilcoxon et al., 2007; Morgan et al., 2013; Buras et al., 2014; Richard et al., 2017), TH transporters (Friesema et al., 2005; Mayerl et al., 2014; Bernal et al., 2015; Groeneweg et al., 2020) and TH deiodinases (Ng et al., 2009, 2010, 2017; Bocco et al., 2016; Stohn et al., 2016, 2018).

In the rodent, it is during the second and third week of life (equivalent to last trimester of gestation in humans) when the brain exhibits most responsiveness to TH (Bernal, 2005). This time coincides with the differentiation of oligodendrocytes and myelination, as well as with peak levels of THs in the serum due to the maturation of the hypothalamic-pituitary thyroid (HPT) axis (Dussault and Labrie, 1975). It also coincides with peak expression of DIO2 in the brain (Bates et al., 1999; Hernandez et al., 2006), the enzyme that enhances TH action by converting thyroxine (T4) into T3, the hormone with highest affinity for the thyroid hormone nuclear receptor (Bianco et al., 2002; St Germain et al., 2009).

However, earlier in development and especially during rodent fetal life, serum TH levels are much lower than in the adult (Dussault and Labrie, 1975). This is due both to the fact that the HPT axis has not attained full functionality and that the fetal tissues and the utero-placental unit express high levels of the type 3 deiodinase (DIO3) (Galton et al., 1999; Huang et al., 2003), the selenoenzyme that clears T4 and T3 by converting them into metabolites with no significant affinity for the nuclear receptor (Bianco et al., 2002; Hernandez, 2005). The low levels of TH during fetal life and the effects of maternal thyroid status on fetal brain development (Richard and Flamant, 2018) have prompted investigators to assess if the fetal brain is responsive to TH. However, the administration of exogenous TH (either T3 or T4) to rat pregnant dams failed to produce responses of two T3 target genes in the embryonic day 21 (E21) brain (Schwartz et al., 1997). In another study, the administration of T3 to hypothyroid rat dams did not regulate three selected responsive genes in the cerebral cortex at fetal ages E17 and E21, while T4 administration exerted a significant effect (Grijota-Martínez et al., 2011). The lack of response to T3 was noted despite abundant expression of TH transporters and receptors. The authors of these studies reached a similar conclusion and suggested the existence of yet unidentified factors that suppress precocious response to T3. These factors may be involved in T3 signaling or in limiting the amount of T3 that reaches target cells (Schwartz et al., 1997; Grijota-Martínez et al., 2011).

We propose that DIO3 is one of such critical factors. Here we used gene expression profiling in DIO3-deficient mice to show that E18.5 *Dio3*−/− fetal brains exhibit significant and broad changes in gene expression. Our results further show that the fetal brain is responsive to TH as early as E13.5 of gestational age and, in the context of previous work, underscore an important role for *Dio3* in protecting the developing brain from premature T3 action.

## Materials and Methods

### Experimental Animals

*Dio3*−/− mice have been previously described (Hernandez et al., 2006). Mice used in the present studies were on a C57Bl/6J genetic background. Original female mice on a 129/SVJ genetic background and heterozygous for the *Dio3* inactivating mutation were mated with wild type C57Bl/6J males for seven generations, and the colony was then maintained for more than 26 generations by interbreeding, and by matings of heterozygous females with commercially obtained C57Bl/6J males every 2–3 years to refresh the colony. Experimental mice used in the present study were *Dio3*+/+ and *Dio3*−/− littermate fetuses generated by timed matings of *Dio3* +/− mice. The morning after mating was considered gestational day E0.5. Dams were euthanized using carbon dioxide asphyxiation at embryonic day 13.5 (E13.5) or E18.5. Uterine horns were placed on iced saline and fetal brains were harvested, frozen on dry ice and kept at −80°C until later processing. Mouse studies were approved by the Institutional Animal Care and Use Committee at Maine Medical Center Research Institute.

### Fetal Brain RNA Sequencing

We performed RNA sequencing on two batches of total RNA samples from whole brains from individual littermate male fetuses at E13.5 (*n* = 4, 2, respectively, for *Dio3* + / + and *Dio3*−/− mice) and E18.5 days of embryonic age (*n* = 3, 5, respectively for *Dio3* + / + and *Dio3*−/− mice). The first batch was submitted to Cofactor Genomics (St. Louis, MO, United States) and sequenced in an Illumina platform. Briefly, rRNA-probes (Ribo-Zero, Epicenter, Madison, WI, United States) were hybridized to total RNA for removal of ribosomal RNA from the sample. Ribo-depleted RNA was then fragmented prior to cDNA synthesis using random primers. Double-stranded cDNA was end-repaired and A-tailed to prepare for adaptor ligation. Indexed adaptors were ligated to DNA, and the adaptor-ligated DNA was amplified by PCR. Library size and quality was assessed on an Agilent Bioanalyzer and library yield was quantified by qPCR using the KAPA Biosystems Library Quantification kit (Kapa Biosystems, Inc., Wilmington, MA, United States) prior to sequencing on (single end, 75 bp fragment size) on the Illumina HiSeq 2000. The number of aligned reads per sample varied between 40 and 50 million, and represented ∼78% of the total reads per sample. Raw sequence data in Fastq format were assessed for quality (FastQC, ^1^) and ribosomal RNA content. Fastq files and processed files for this experiment have been deposited on the Gene Expression Omnibus (GEO) database (Accession number GSE172000). Libraries for a second batch of samples were prepared using a NEBNext rRNA Depletion Kit (New England Biolabs, #6310) to deplete ribosomal RNA from the total RNA. Then, a NEBNext Ultra II RNA Library Prep Kit for Illumina (New England Biolabs, #E7770) was used to construct the RNA-seq library. The quality and quantity of input RNA and the libraries were assessed using an Agilent Bioanalyzer and Qubit. The multiplex libraries were sequenced (single end, 75 bp fragment size) on a Next Gen 550 at Tufts University Genomics Core Facility.

### RNA Sequencing Data Analyses

FASTQC (see text footnote 1) and multiQC v1.9 (Ewels et al., 2016) were used to determine the quality of sequencing data for all samples. Adaptor sequences were trimmed using Cutadapt 3.1 with Python 3.6.2 and the -m 1 option (^2^ STAR) (Spliced Transcripts Alignment to a Reference, version 2.5.3a) (Dobin et al., 2013) was used to align the reads to the reference genome (GRCm38/mm10) using the default settings, and the genome index was built based on the GENCODE VM22 annotation (GRCm38/mm10). rRNA and tRNA were filtered using bedtools version 2.26.0, using the GTF files downloaded from the UCSC Table Browser (GRCm38/mm10). HTSeq (Anders et al., 2015) was used to count the reads per transcript (HTseq counts) with a default setting over the union with -s *no* option. For differential gene expression analysis, DESeq2 version 1.30.1 (Love et al., 2014) was performed in the R environment (Version 4.0.3), using the HTseq counts and the significance cutoff set by default to an adjusted *P* < 0.05. ComBat-seq (Zhang et al., 2020) was utilized to adjust for batch effects between the Cofactor Genomics and Tufts University sequencing datasets. Heatmap, MA, PCA, UpSet (Conway et al., 2017) plots were created in R, based on both coding and non-coding transcripts. Functional ontology and pathway analyses of differentially expressed genes were performed using Ingenuity Pathway Analysis (IPA) software (Qiagen, Valencia, CA, United States), and the Database for Annotation, Visualization and Integrated Discovery (DAVID)^3^.

### Real Time Quantitative PCR

Fetal brains were harvested and subsequently frozen on dry ice, and total RNA was extracted using the RNeasy kit from Qiagen (Valencia, CA, United States). Total RNA (1 μg) was reverse transcribed with M-MLV reverse transcriptase in the presence of random decamers (both from Thermo Fisher Scientific, Waltham, MA, United States) at 65°C for 5 min, then 37°C for 50 min. The 20 μl reverse transcription reactions were diluted by adding 230 μl DNase and RNase free water. An aliquot of each sample was mixed together for an internal standard and diluted fourfold. Real-time PCR reactions were set up in duplicate with gene-specific primers and SYBR Select Master Mix (Thermo Fisher Scientific, Waltham, MA, United States) and run on the CFX Connect from Bio-Rad (Hercules, CA, United States), where they underwent an initial 10 min denaturing step, followed by 36 cycles of a denaturing step (94°C for 30 s) and an annealing/extension step (60°C for 1 min). For each individual sample, expression was corrected by the expression of control, housekeeping genes (*Gapdh* or *Rn18s)*, which did not exhibit any significant difference in expression between genotypes. Expression data are shown in arbitrary units and represented as fold-increase over the mean value in the control group. The sequences of the primers used for each gene are shown in Supplementary Table 1.

### RNAscope *in situ* Hybridization

The heads of E18.5 fetuses were harvested as described above and fixed in 4% formaldehyde for 48 h. After fixation they were paraffin-embedded and cut in five microns coronal or rostro-caudal sections. *In situ* hybridization of *Klf9* and *Nrgn* mRNAs was performed in selected sections of two animals per genotype utilizing the RNAscope technique (Advanced Cell Diagnostics, BioTechne Corporation, Newark, CA, United States) following the manufacturer’s suggested procedures. We used the RNAscope Mm-Klf9 and Mm-Nrgn probes (catalog numbers 488371 and 499441, respectively) and the ACD 2.5HD Detection kit (RED). As a negative control, we used the bacterial probe DapB supplied by the manufacturer. Some tissue sections were counterstained with hematoxyline and mounted with EcoMount (catalog # EM897L, Biocare Medical, Pacheco, CA, United States), while other sections were mounted with DAPI Fluoromount-G (Catalog # 0100-20, Southern Biotech, Birmingham, AL, United States). Bright field or fluorescent images of the mRNA signal were taken, respectively, with a Zeiss Axioskop 40 microscope or a Leica SP8 confocal microscope utilizing LAS X software. For anatomic reference, adjacent tissue sections were stained with H&E at our Histology Core facility following standard procedures.

### Statistical Analyses

Statistical analysis of data other than RNA-sequencing data was performed using the statistical tools of GraphPad Prism 6 (GraphPad Software, Inc.). A Student’s *t*-test, and one-way ANOVA or two-way ANOVA followed by Tukey’s test were used to determine statistical significance, which was defined as *P* < 0.05. Significance between different distribution frequencies of genes was determined using a standard chi-square test.

## Results

### Expression of Genes Related to Thyroid Hormone Action in the Fetal Brain

**FIGURE 1:**
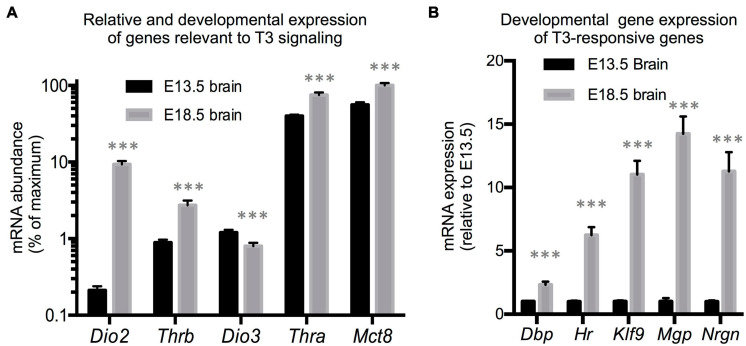
E13.5 and E18.5 brain gene expression. **(A)** Developmental brain expression of genes that regulate T3 action. The relative abundance of mRNA between different genes is an estimation based on CT values on the assumption of similar annealing efficiency of primers used in real time qPCR. **(B)** Developmental brain gene expression of selected T3-responsive genes. Data represent the mean ± SEM of **(A)** eight different brains per experimental group representing both sexes and five different litters or **(B)** 8 (E13.5) and 11 (E18.5) different brains representing mice of both sexes and 6 (E13.5) or 8 (E18.5) litters. ^∗∗∗^*P* < 0.001 E13.5 vs E18.5 as determined by the Student’s *t*-test.

We first utilized RNA samples isolated from wild type brains from fetuses at E13.5 and E18.5 of gestational age to evaluate the expression of genes with a prominent role in determining TH action. Assuming comparable efficiency in the primers used for each gene, the most abundant mRNAs in E13.5 brains were estimated to be those encoding for the preferred T3 transporter MCT8 (*Slc16a2*) and for TH receptor alpha 1 isoform (THRA1) (Figure 1A). Notably less abundant were *Dio3* and TH receptor beta (*Thrb1* isoform) mRNAs, while *Dio2* mRNA was the least abundant. Between E13.5 and E18.5 of gestational age, brain expression of *Dio3* significantly decreased, while the expression of *Mct8*, *Thra*, and *Thrb* was increased, with *Mct8* and *Thra* mRNAs still being the most abundant (Figure 1A). Particularly notable was the 50-fold developmental increase in *Dio2* mRNA abundance. The developmental increases in T3 transporter, receptors and T3-generating DIO2 enzyme suggest that the E18.5 brain should exhibit increased T3 signaling compared to the E13.5 brain. We thus measured the expression of five well-established T3-responsive genes in the brain (Chatonnet et al., 2015) at both developmental ages. We observed significant developmental increases in the expression of Krüppel-like factor 9 (*Klf9*), hairless (*Hr*), Neurogranin (*Nrgn*), D site albumin promoter binding protein (*Dbp*) and Matrix gla protein (*Mgp*) (Figure 1B), supporting the hypothesis that the mechanisms controlling T3 action are more mature in the E18.5 brain than earlier in gestation.

### Expression of *Klf9* and *Nrgn* in the *Dio3*−/− E18.5 Brain

**FIGURE 2:**
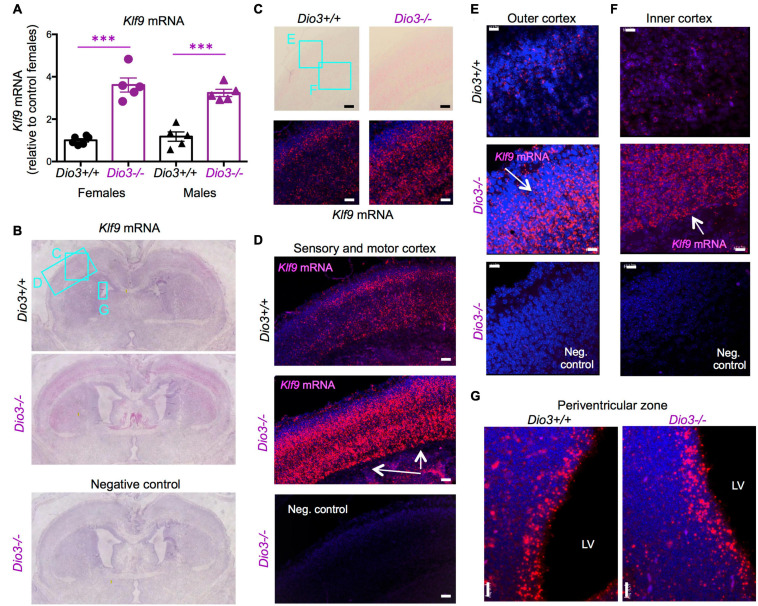
*Klf9* expression is elevated in *Dio3*−/− brains. **(A)** *Klf9* mRNA expression in whole E18.5 brains. Data represent the mean ± SEM of 11 and 10 different samples from E18.5 *Dio3*+/+ and Dio3−/− mice, respectively, divided by sex. ^∗∗∗^*p* < 0.001 E13.5 vs E18.5 as determined by the ANOVA and Tukey’s *post hoc* test. B-G, Bright field and fluorescent images of *in situ* hydridization of *Klf9* mRNA in whole brain coronal sections **(B)**, motor cortex **(C)**, motor and sensory cortex **(D)**, outer and inner cortical layers [**(E,F)**, respectively] and lateral periventricular zone **(G)**. Images are representative of two different animals of each genotype. Rectangles and letters indicate the panels in which those anatomic regions are amplified. St, striatum; Lv, lateral ventricle. Negative control was hybridized with a bacterial probe. Arrows indicate major areas of differential expression. Scale bars, 45, 15, and 12 microns for panels **(C**–**G)**, respectively.

Based on the data above, and since we have previously shown that serum T3 is elevated in *Dio3*−/− fetuses late in gestation (Hernandez et al., 2006), we focused on E18.5 developmental stage to evaluate T3-dependent gene expression in the *Dio3*−/− brain. We chose *Klf9* and *Nrgn* for these studies. Real time qPCR analysis of RNA from whole E18.5 *Dio3*−/− brains indicated a more than threefold increase in *Klf9* expression when compared with that of *Dio3*+/+ littermates (Figure 2A). We observed no indication of sexual dimorphisms in *Klf9* expression in either *Dio3*+/+ or *Dio3*−/− fetuses. *In situ* hybridization using RNAscope revealed that the increase is apparent in most cortical and striatal areas (Figure 2B). *Klf9* was strongly expressed in the neocortex, except for the most external layer (Figure 2C). Compared to *Dio3*+/+ littermates, the *Dio3*−/− brain exhibited robust increases in *Klf9* expression across multiple areas of the neocortex (Figure 2C), including the motor and sensory cortices (Figure 2D) and both external and deeper cortical layers in which *Klf9* was expressed (Figures 2E,F, respectively). Marked increases in *Klf9* mRNA were also observed in the *Dio3*−/− septum and striatum (Supplementary Figures 1B,C, respectively). Elevated *Klf9* mRNA was more modest in the periventricular zone of the third ventricle (Supplementary Figures 1E,F). Interestingly, no apparent changes in *Klf9* expression were observed in the periventricular zone of the lateral ventricles (Figure 2G and Supplementary Figure 1C).

**FIGURE 3:**
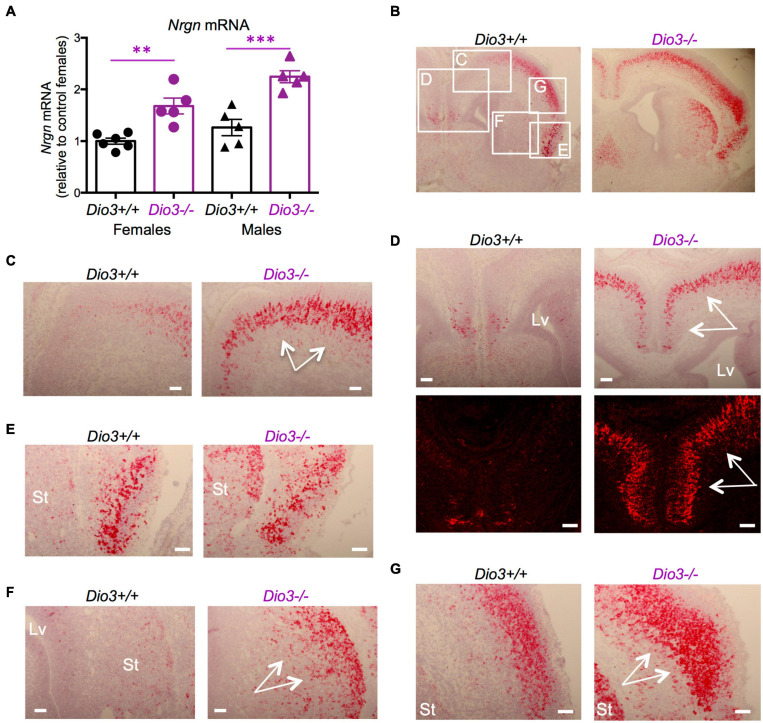
*Nrgn* expression is elevated in E18.5 *Dio3*−/− brains. **(A)** *Nrgn* mRNA expression in whole E18.5 brains. Data represent the mean ± SEM of 11 and 10 different samples from E18.5 Dio3+/+ and Dio3−/− mice, respectively, divided by sex. ^∗∗^and ^∗∗∗^*p* < 0.01 and *p* < 0.001, respectively, E13.5 vs E18.5 as determined by the ANOVA and Tukey’s *post hoc* test. **(B–G)**, Bright field and fluorescent images of *in situ* hydridization of *Nrgn* mRNA in whole brain coronal sections **(B)**, motor cortex **(C,D)**, frontal/cingular cortex **(D)**, piriform cortex **(E)**, striatum **(F)** and second somatosensory cortex **(G)**. Images are representative of two different animals of each genotype. Rectangles and letters indicate the panels in which those anatomic regions are amplified. St, striatum; Lv, lateral ventricle. Arrows indicate areas of major differential expression. Scale bars are 40 microns.

The expression of *Nrgn* was also significantly elevated in the E18.5 *Dio3*−/− brains compared to that of littermates, both in males and females (Figure 3A). *In situ* hybridization indicated elevated *Nrgn* expression in most brain regions (Figure 3B and Supplementary Figure 2A). *Nrgn* expression increase in *Dio3*−/− fetuses was most dramatic in the frontal/cingular cortex (Figure 3D and Supplementary Figure 2B) as well as in the motor cortex (Figure 3C) and striatum (Figure 3F). *Nrgn* expression was also elevated in the motor cortex (Figure 3G), but no appreciable change was observed in the piriform cortex (Figure 3E). Similarly to *Klf9*, no *Nrgn* expression was noted in the most outer layer of the cortex (Supplementary Figure 2B). The hematoxylin counterstaining and the pattern of cortical *Nrgn* expression suggested increased brain cortical thickness in *Dio3*−/− fetuses (Supplementary Figure 2B).

### Gene Expression Profiling of the *Dio3*−/− Fetal Brain

**FIGURE 4:**
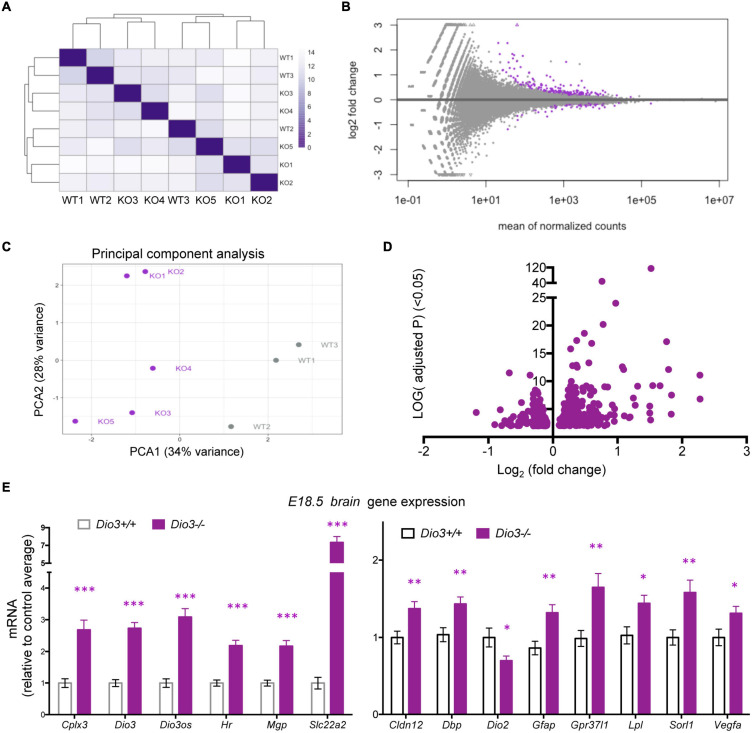
Gene expression profiling of E18.5 *Dio3*+/+ and *Dio3*−/− male brains. **(A)** heat map representing the expression and clustering of the RNA samples used. **(B**–**D)** MA, PCA, and volcano plots showing 588 DEGs (adjusted *P* < 0.05) in purple color. In PCA panel, WT1-3 and KO1-5 represent samples from *Dio3*+/+ and *Dio3*−/− mice. **(E)** qPCR validation of selected DEGs in an extended number of samples, including females. Data represent the mean ± SEM relative to control mean value of 11 and 10 different samples from E18.5 *Dio3*+/+ and *Dio3*−/− mice. ^∗^, ^∗∗^, and ^∗∗∗^, indicate *p* < 0.05, *p* < 0.01, and *p* < 0.001, respectively, E13.5 vs E18.5 as determined by the Student’s *t*-test. (Note: *Dio3*−/− mice express *Dio3* mRNA, although they carry an inactivation mutation of the coded protein).

Results on *Klf9* and *Nrgn* expression suggested that the E18.5 brain is sensitive to TH and that mouse DIO3 deficiency is an excellent model to probe T3-dependent gene expression in the early development of the brain. Thus, we used RNA sequencing to perform a gene expression profiling of three *Dio3*+/+ and five *Dio3*−/− male brains at E18.5 of embryonic age. Principal component analysis (PCA) distinctively separated *Dio3*+/+ and *Dio3*−/− samples along PC1 (34% variance) but not along PC2 (28% variance) (Figure 4C). One of the *Dio3*+/+ samples clustered within the *Dio3*−/− samples (Figure 4A). We identified 246 differentially expressed genes (DEGs) with an adjusted *P* < 0.05 that are represented in MA and volcano plots in Figure 4 (Figures 4B,D, respectively). [599 genes were differentially expressed based on a non-adjusted *P* < 0.01 (Supplementary Data)]. DEGs showed a marked bias toward up-regulation, as 171 genes (70% of DEGs at that statistical threshold) (Figure 4D). Differential expression of some DEGs was confirmed by qPCR using the same plus additional, non-related samples from males and female E18.5 fetuses. Strong up-regulation was validated for the expression of *Cplx3, Dio3* itself, *Dio3os, Hr, Mgp*, and *Slc22a2* (Figure 4E, left) [please note that *Dio3*−/− mice carry a triple point mutation in *Dio3* that renders the DIO3 enzyme fully inactive, but *Dio3* mRNA is present and detectable in these animals (Hernandez et al., 2006)]. Significant up-regulation was also confirmed for the expression of *Cldn12, Dbp, Gfap, Gpr37l1, Lpl, Sorl1*, and *Vegfa*, while the expression of *Dio2* was modestly repressed (Figure 4E, right).

**FIGURE 5:**
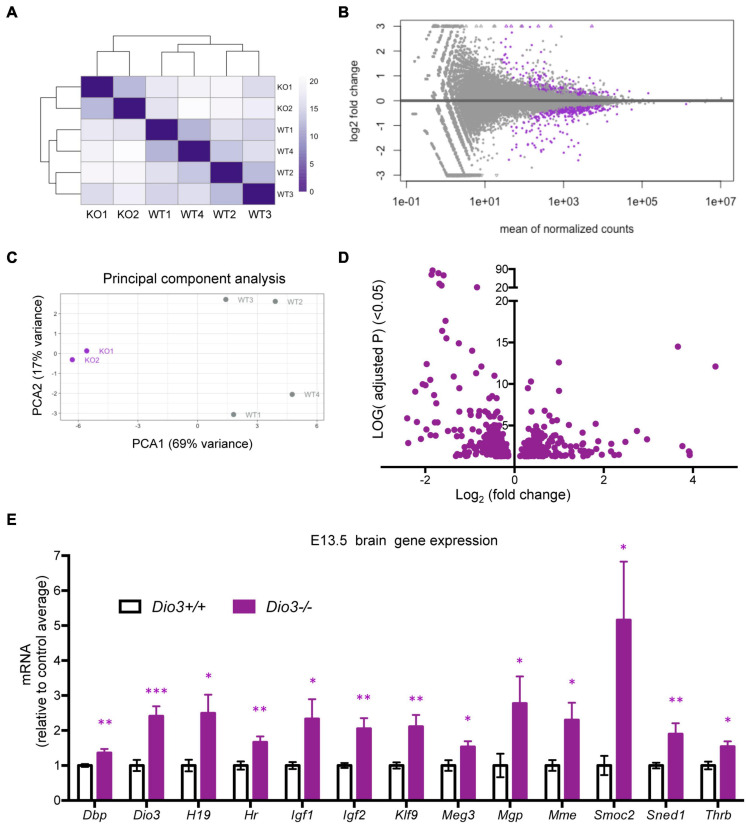
Gene expression profiling of E13.5 *Dio3*+/+ and *Dio3*−/− male brains. **(A)** Heat map representing the expression and clustering of the RNA samples used. **(B–D)** MA, PCA, and volcano plots showing 588 DEGs (adjusted *P* < 0.05) in purple color. In PCA panel, WT1-4 and KO1-2 represent samples from *Dio3*+/+ and *Dio3*−/− mice. **(E)** qPCR validation of selected DEGs in an extended number of samples, including females. Data represent the mean ± SEM relative to control mean value of 8 and 6 different samples from E18.5 *Dio3*+/+ and *Dio3*−/− mice. ^∗^, ^∗∗^, and ^∗∗∗^, indicate *p* < 0.05, *p* < 0.01, and *p* < 0.001, respectively, E13.5 vs E18.5 as determined by the Student’s *t*-test. (*Dio3*−/− mice express *Dio3* mRNA, although they carry an inactivation mutation of the coded protein).

To investigate whether the brain capable of responding to T3 even earlier in development, we also submitted for RNA sequencing E13.5 brain RNA samples from four *Dio3*+/+ and two *Dio3*−/− males. Sample clustering and MA plot highlighting DEGs based on an adjusted *P* < 0.05 are shown in Figures 5A,B. PCA robustly separated *Dio3*+/+ and *Dio3*−/− samples along PC1 (69% of variance), but not along PC2 (17% of variance) (Figure 5C). Samples of different genotypes clustered separately (Figure 5A). At this gestational age, we identified 588 DEGs based on an adjusted *P* < 0.05. At this age, there was a bias toward down-regulation, with 383 (65%) of DEGs being down-regulated (Figure 5D). [1,012 differentially expressed based on a non-adjusted *P* < 0.01 (Supplementary Data)]. Using the same plus additional RNA samples from female E13.5 brains, we used qPCR and confirmed the differential expression of some genes consistently found to be up-regulated by T3 including *Dbp, Dio3, Klf9, Mgp, Mme, Sned1*, and *Thrb* (Figure 5E). We also confirmed up-regulation of genes with important developmental roles including *Igf1, Igf2, H19*, and *Meg3* (Figure 5E). Overall, these results show that the fetal brain is sensitive to T3 as early as embryonic age E13.5.

We used DAVID and IPA to analyze the ontology and biological functions of DEGs at each gestational age. To avoid bias due to differences in the number of genes entered into these algorithms, we used 588 DEGs at E13.5 (adjusted *P* < 0.05) and 599 DEGs at E18.5 (non-adjusted *P* < 0.01). For each dataset statistically enriched terms with a FDR < 0.0001 are listed in the Supplementary Data. Selected enriched terms identified by DAVID are shown for E13.5 and E18.5 DEGs in Tables 1, 2, respectively. There were enriched biological themes common for both sets of DEGs showing comparable statistical significance, including “neurogenesis,” “methylation,” extracellular matrix,” “alternative splicing,” “activator,” “repressor,” “differentiation.” Enrichment terms including “glycoprotein” and “EGF-like” were more significant or specific for DEGs at E18.5. Other enrichment terms were much more significant or specific for DEGs at E13.5, including “phosphoprotein,” “developmental protein,” ”transcriptional regulation,” “DNA binding,” “nucleus,” chromosome,” “homeobox,” and “cell cycle” (Tables 1, 2).

**TABLE 1:** Results of DAVID analysis of DEGs in E13.5 *Dio3*−/− brain.

UP_KEYWORD Term	Fold Enrichment	FDR
DNA-binding*	4.68	5.36E-70
Nucleus	2.52	9.79E-55
Chromosome	7.11	9.43E-36
Developmental protein*	4.32	7.40E-34
Phosphoprotein*	1.69	1.83E-27
Homeobox	7.53	9.16E-27
Nucleosome core	13.49	7.86E-25
Cell cycle	4.38	9.24E-22
Ubl conjugation*	2.91	1.14E-21
Transcription regulation*	2.67	5.50E-21
Transcription*	2.61	2.13E-20
Mitosis	6.35	3.48E-18
Cell division	5.10	2.11E-17
Citrullination	11.21	2.83E-17
Isopeptide bond*	3.05	6.26E-15
DNA replication	10.21	6.26E-15
Acetylation	1.88	1.21E-12
Activator*	3.31	1.07E-11
Methylation*	2.59	8.08E-10
Neurogenesis*	4.44	1.40E-08
Alternative splicing*	1.47	4.55E-07
Extracellular matrix*	4.13	5.19E-07
Differentiation*	2.55	2.97E-06
Repressor*	2.68	6.03E-06
Centromere	5.03	6.50E-06
Cytoskeleton	1.96	8.19E-05
Glycoprotein*	1.44	8.71E-05
Microtubule	3.19	1.58E-04
Zinc	1.59	3.38E-04
Cyclin	8.03	4.10E-04
LIM domain	5.70	4.63E-04
Kinetochore	4.88	6.18E-04
DNA repair	2.87	9.10E-04

Based on analysis of 588 DEGs based on an adjusted *P* < 0.05. * indicates a common term for both E13.5 and E18.5 DEGs.

**TABLE 2:** Results of DAVID analysis of DEGs in E18.5 *Dio3*−/− brain.

UP_KEYWORD Term	Fold Enrichment	FDR
Glycoprotein*	2.067362941	8.62E-24
Disulfide bond	1.959425632	2.64E-15
Alternative splicing*	1.650343088	1.29E-12
Phosphoprotein*	1.452714812	1.44E-12
Cell adhesion	3.761449216	9.84E-12
Signal	1.641066106	9.84E-12
Developmental protein*	2.492625787	3.55E-09
EGF-like domain	4.55449827	2.13E-08
Secreted	2.002690132	2.60E-08
Neurogenesis*	4.289256546	2.77E-08
Activator*	2.766835241	9.03E-08
Transcription regulation*	1.875782586	4.01E-07
Transcription*	1.857455826	4.01E-07
DNA-binding*	1.883655912	1.98E-06
Extracellular matrix*	3.840388721	2.89E-06
Methylation*	2.166306228	4.29E-06
Differentiation*	2.429644231	9.75E-06
Cell membrane	1.471844735	1.50E-05
Calcium	2.182566746	2.06E-05
Synapse	2.857724405	7.25E-05
Ubl conjugation*	1.746841555	1.33E-04
Metal-binding	1.444725859	1.61E-04
Repressor*	2.351386027	2.17E-04
Glycolysis	8.968858131	2.55E-04
GPI-anchor	3.923875433	6.20E-04
Membrane	1.206581528	6.20E-04
Cell junction	2.07769501	8.56E-04
Isopeptide bond*	1.852826804	9.98E-04

Based on analysis of 599 DEGs based on non-adjusted *P* < 0.01. * indicates a common term for both E13.5 and E18.5 DEGs.

Ingenuity pathway analysis analysis of the two DEGs datasets indicated enrichment in a number of canonical pathways, some of which were common (Supplementary Data). However, the most statistically significant canonical pathways were very different between the two developmental stages (Table 3). While E13.5 DEGs were most enriched in pathways related to the cell cycle and nuclear DNA rearrangement during mitosis, E18.5 DEGs showed top enrichment in pathways related axon guidance, synaptogenesis, glycolysis and hypoxia inducible factor (Table 3 and Supplementary Data).

**TABLE 3:** Top canonical pathways (IPA) enriched in E13.5 and E18.5 DEGs.

Canonical Pathway	-LOG(*P* value)	Age
Cell Cycle Control of Chromosomal Replication	12.1	E13.5
Aryl Hydrocarbon Receptor Signaling	8.5	E13.5
PCP pathway	7.15	E13.5
Kinetochore Metaphase Signaling Pathway	7.09	E13.5
Mitotic Roles of Polo-Like Kinase	5.75	E13.5
Wnt/β-catenin Signaling	5.13	E13.5
Axonal Guidance Signaling	8.26	E18.5
HIF1α Signaling	6.56	E18.5
Glycolysis I	5.61	E18.5
Synaptogenesis Signaling Pathway	4.41	E18.5
Hepatic Fibrosis/Hepatic Stellate Cell Activation	3.97	E18.5
TR/RXR Activation	3.63	E18.5

**FIGURE 6:**
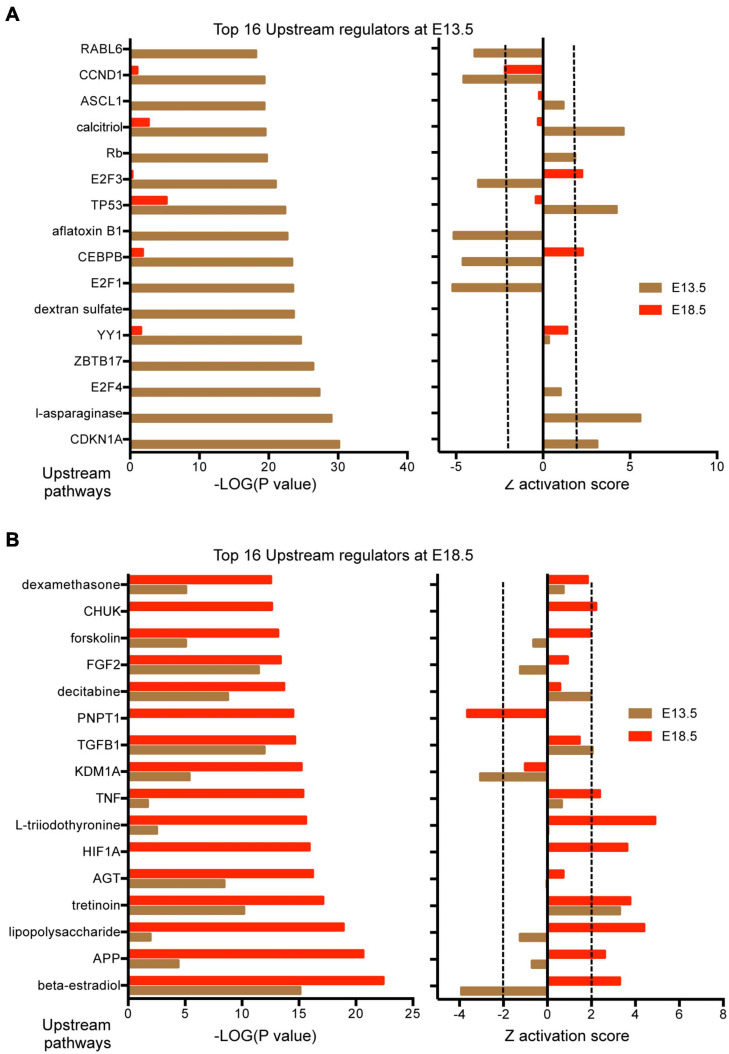
Upstream regulators affected by DEGs in the E13.5 and E18.5 brains of *Dio3*+/+ and Dio3−/− mice. **(A)** Top significant upstream pathways affected in Dio3−/− mice at E13.5 with corresponding statistical values and Z activation scores at E18.5. **(B)** Top significant upstream pathways affected in *Dio3*−/− mice at E18.5 with the corresponding statistical values and Z activation scores at E13.5. Dotted lines indicate the Z score threshold hat IPA considers significant for activation or inactivation of a certain pathway.

**FIGURE 7:**
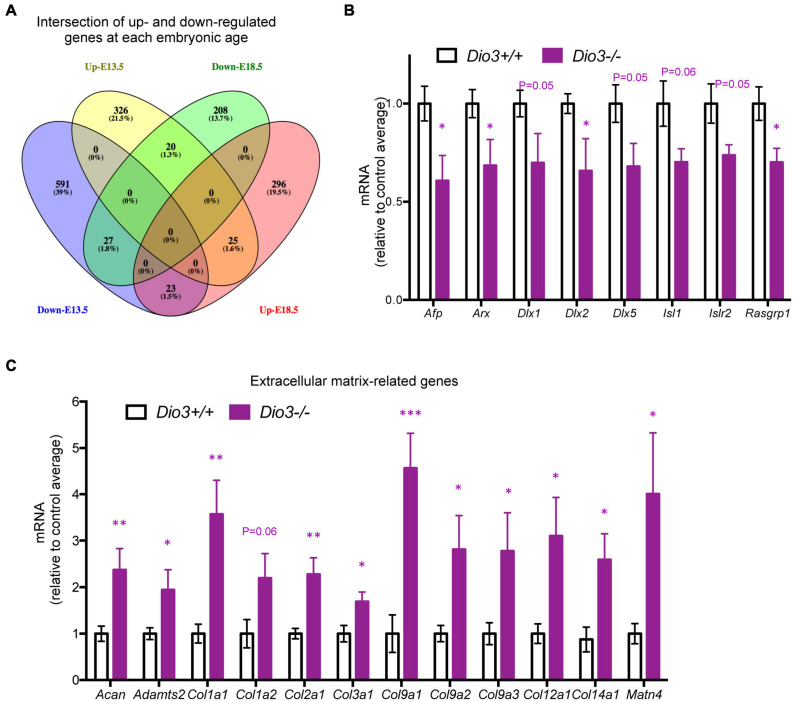
Overlap and differential gene expression in E13.5 Dio3−/− brains. **(A)** Venn diagram of up- and down regulated genes in Dio3−/− brain at E13.5 and E18.5. **(B)** qPCR validation of additional DEGs that are down-regulated in E13.5 *Dio3*−/− brains. **(C)** qPCR validation of differential expression of genes related to extracellular matrix components in E13.5 *Dio3*−/− brains. Data represent the mean ± SEM, relative to control mean value, of 8 and 6 different samples from E18.5 *Dio3*+/+ and *Dio3*−/− mice. ^∗^, ^∗∗^, and ^∗∗∗^, indicate *p* < 0.05, *p* < 0.01, and *p* < 0.001, respectively, E13.5 vs E18.5 as determined by the Student’s *t*-test.

Results from IPA concerning pathway upstream analysis rendered substantially different results. The most significant upstream regulators identified from E13.5 DEGs, including CDKN1A, asparaginase and E2F4, barely showed any significance for E18.5 DEGs, an observation that also applies to the activation Z scores associated with those regulators (Figure 6A). The activation Z scores and the effects of these regulators, as well as the main canonical pathways affected as revealed by IPA (Supplementary Data) suggest a reduction in cell proliferation, consistent with the results from DAVID. In contrast, a substantial proportion of the top significant upstream pathways affected by DEGs at E18.5 were also affected at E13.5 with comparable significance, including those regulated by beta-estradiol, FGF2, TGFB1, AGT, and tretinoin, a retinoic acid agonist (Figure 6B). Interestingly, some of these regulators exhibited similar (tretionein, TGFB1) or opposite (beta-estradiol, FGF2) activation scores at each developmental stage (Figure 6B). Furthermore, although the overlap of DEGs at both E13.5 and E18.5 was significant (95 genes of 1,012 and 599, respectively), it was substantially lower than anticipated if we consider the hypothesis that the effects of T3 on the fetal brain are largely comparable at both gestational ages. A large majority of DEGs were not common between E13.5 and E18.5 brains and about half of the common DEGs showed opposite regulation between developmental ages (Figure 7A). Using additional samples from female fetuses, we used qPCR to validate the differential expression of E13.5 DEGs. The differential expression of some of them was borderline significant. These DEGs included Arx, *Dlx1, Dlx2, Dlx5, Isl1*, and *Islr2* (Figure 7B), which are of importance for neuronal and cortical development. We also confirmed the differential expression at E13.5 of several genes related to collagen and extracellular matrix formation (Figure 7C). Taken together,

these observations suggest common biological processes in the brain affected by T3 at both developmental ages, but also indicate that some of them are distinct and specific to E13.5.

An initial analysis of gene expression profiles between developmental ages within the same genotype identified 9,408 DEGs (adjusted *P* < 0.01) in the *Dio3*+/+ brain between E13.5 and E18.5. In *Dio3*−/− mice, 3,841 genes were identified as differentially expressed between developmental ages, the vast majority of them (3,128 genes, 81%) overlapping with those in *Dio3*+/+ mice (Supplementary Figure 3). These results indicate that there are more than five thousand genes that showed significant changes in brain expression during development in *Dio3*+/+ mice but not in *Dio3*−/− mice (Supplementary Figure 3).

## Discussion

The importance of TH for the development of the CNS is well established in mammals, including humans (Legrand, 1984). In rodents, their broader and more profound effects on the regulation of brain gene expression occur in late neonatal life (Bernal, 2005; Morte et al., 2010; Hernandez et al., 2012), but their actions at earlier developmental stages, especially *in utero*, have remained unclear. The administration of TH to pregnant rodents in late gestation has minimal or negligible effects on the expression of T3-regulated genes (Schwartz et al., 1997; Grijota-Martínez et al., 2011), despite the relatively abundant expression in the fetal brain of the T3 receptor THRA and the main T3-transporter, MCT8 (López-Espíndola et al., 2014; Mayerl et al., 2014). To determine whether the fetal brain is sensitive to T3, here we used a DIO3-deficiency mouse model, in which an excess of T3 in the fetus is produced by impaired T3 clearance.

Developmental expression profiles of genes enhancing brain T3 availability and action, as well as selected genes regulated by T3 (Chatonnet et al., 2015), showed significantly increased expression at E18.5 compared to E13.5, suggesting that components of T3 signaling are more mature at the later fetal age. Using qPCR and *in situ* hydridization we showed that *Dio3*−/− mice exhibited robust mRNA up-regulation of *Klf9* and *Nrgn*, two well-established T3-regulated genes (Martínez de Arrieta et al., 1999; Chatonnet et al., 2015) that have been shown to be regulated in primary culture of fetal neurons (Gil-Ibanez et al., 2014) and in different regions of the neonatal and adult brain (Iñiguez et al., 1992; Martínez de Arrieta et al., 1999). The expression of both *Klf9* and *Nrgn* was increased in most brain regions of E18.5 *Dio3*−/− mice in which they were expressed, predominantly areas of the cortex and striatum, although *Nrgn* manifested region-specific sensitivity to thyroid hormones, as previously described in older animals (Guadaño-Ferraz et al., 1997), with most prominent up-regulation in the frontal cortex and striatum.

Unbiased gene expression profiling by RNA sequencing confirmed the sensitivity of the *Dio3*−/− fetal brain to T3 by identifying, with a limited sample number, several hundred differentially expressed genes. Many of these genes are included in a published compendium of T3 regulated genes in the CNS (Chatonnet et al., 2015), suggesting that T3-regulation of gene expression in the brain at this developmental age is largely comparable to that later in life in terms of the genes that are regulated. Canonical pathways enriched in DEGs as identified by IPA and DAVID, including axon guidance signaling and synaptogenesis are consistent with known TH effects in the developing brain (Bernal, 2005). Activation of glycolysis-, hypoxia- and inflammation-related pathways (HIF1a and lipopolysaccharide) may reflect the action of T3 excess on oxidative-dependent metabolism and the brain cell response to reduced T3 levels, as HIF1a is known to activate *Dio3* expression in the brain (Simonides et al., 2008). One of the top canonical pathways and upstream regulators identified by IPA as being activated is that of thyroid receptor-retinoid X receptor (“TR-RXR”) and “triiodothyronine,” providing further confirmation of enhanced T3 signaling. The significant activation of upstream regulators (tretionein, dexamethasone) related to other nuclear receptors also suggest the occurrence of cross-talk between TH signaling and pathways regulated by the retinoic acid and glucocorticoid receptors, something that has been proposed in a model of primary culture of fetal neurons (Gil-Ibanez et al., 2014).

At E13.5, we also observed and validated the increased expression of well-established T3-regulated genes including *Dbp, Dio3, Hr, Klf9, Mgp, Mme, Sned1*, and *Thrb.* This finding indicated that the brain is sensitive to T3 as early as E13.5. However, a large proportion of DEGs at this embryonic age is substantially different from those identified at E18.5, suggesting largely different biological effects. This is illustrated by the rather low overlap in DEGs between both ages, and further confirmed by the different biological terms and pathways identified by DAVID and IPA as achieving top statistical significance. Both DAVID and IPA algorithms suggest E13.5 DEGs been involved in the cell cycle. In particular, upstream regulators identified by IPA indicate negative activation scores of pathways or compounds promoting transcription related to the cell cyle (RABL6, CCDN1, E2F3, E2F1, CEBPB, and aflatoxin) (Leone et al., 1998; Piva et al., 2006; Bryant et al., 2020; Huang et al., 2020), and positive activation scores for pathways opposing cell division (TP53, asparaginase, and CDKN1A) (Scotti et al., 2010; Lüdtke et al., 2013; Fischer et al., 2016; Yang et al., 2017). However, some genes like *E2f3* and *E2f1*, with primary functions regulating the cell cycle also play roles in the migration (McClellan et al., 2007) and apoptosis of neurons (Hou et al., 2000) and in neurogenesis (Cooper-Kuhn et al., 2002). These analyses suggest an effect of T3 in suppressing brain cell proliferation or influencing neuronal homeostasis at this embryonic stage. Since most of the DEGs at this developmental stage do not overlap with known T3-target genes in the CNS (Chatonnet et al., 2015), it is possible that they are not primary targets of T3. The differential expression observed may be secondary to changes in cellular subtypes characteristics and population. It is possible that at this early stage T3 targets a particular cell type that in turn will affect gene expression in other cells in a paracrine manner. This possibility is supported by the increased expression of genes involved in the composition of the extracellular matrix, including several collagen genes.

An interesting observation is that IPA identifies beta-estradiol as an upstream regulator whose pathway is significantly altered at both developmental stages. However, the activation score is completely the opposite, with the beta-estradiol pathways being markedly suppressed at E13.5 and activated at E18.5 in *Dio3*−/− fetuses. Furthermore, in E13.5 brains, the strong statistical significance of the differential expression of some genes (*Dlx1, Dlx2, Dlx5, Arx, and Isl1*) in the RNA sequencing experiment (which used only male samples) was barely achieved in the qPCR determinations, which also included female samples. This raises the possibility of a potential sexually dimorphic effect of T3 on the brain in early development that need further investigations, especially since some of the above genes are critically involved in neuronal specification and cortical development (Pla et al., 1991; Eisenstat et al., 1999; McKinsey et al., 2013; Erb et al., 2017; Zhang et al., 2018). It is interesting to note that genes of the *Dlx* family, which are abnormally expressed in the E13.5 *Dio3*−/− brain, influence the development of interneurons (Long et al., 2009). Some of them will later develop into parvalbumin-positive neurons, a known target of T3 in the mature brain (Sui et al., 2007; Mittag et al., 2013; Bastian et al., 2014), suggesting a thyroid hormone developmental programming of the adult brain in terms of T3 responsiveness and T3-dependent brain functions.

Despite the DEGs identified at both gestational ages as regulated by T3, their number is modest compared to those that are regulated by developmental age in either genotype. Yet there are more than 5K genes that show a developmental difference in expression in *Dio3*+/+ mice but do show a developmental change in *Dio3*−/− mice. This number of genes is much larger than those differentially expressed between genotypes at either developmental age. This divergence suggests that for many genes, although differential expression does not achieve statistical significance at a given age, their expression trajectory during development is modified by DIO3 deficiency, an interesting possibility that requires further analyses.

In summary, we show broad differences in gene expression in the brain of fetuses with DIO3 deficiency, demonstrating that the fetal brain is sensitive to T3. The model used further indicates that *Dio3* is a critical modulator of this sensitivity and probably the main reason why other models of altered thyroid hormone status based on TH administration have shown very limited effects. Future research using this model may provide additional insights into the role of TH in early brain development.

## Data Availability Statement

RNA-sequencing fastq and processed files are publicly available at Gene Expression Omnibus (GEO) Database (Accession number: GSE172000).

## Ethics Statement

The animal study was reviewed and approved by Maine Medical Center Research Institute Institutional Animal Care and Use Committee.

## Author Contributions

MM performed the animal work, RNAScope, and real time PCR experiments, drafted the corresponding methods, results, and figures, and edited the manuscript. AH designed the study, analyzed RNA sequencing results, and wrote the manuscript. Both authors contributed to the article and approved the submitted version.

## Conflict of Interest

The authors declare that the research was conducted in the absence of any commercial or financial relationships that could be construed as a potential conflict of interest.

## Footnotes

1 http://www.bioinformatics.babraham.ac.uk/projects/fastqc/

2 https://doi.org/10.14806/ej.17.1.200

3 https://david.ncifcrf.gov/
