# Supplementary material for: The Type 3 Deiodinase Is a Critical Modulator of Thyroid Hormone Sensitivity in the Fetal Brain
Source: Front Neurosci. 2021 Jun 29;15:703730. doi: 10.3389/fnins.2021.703730 (PMC8265566; doi:10.3389/fnins.2021.703730)
Supplement: Supplementary file 3 [file Data_Sheet_1.PDF]

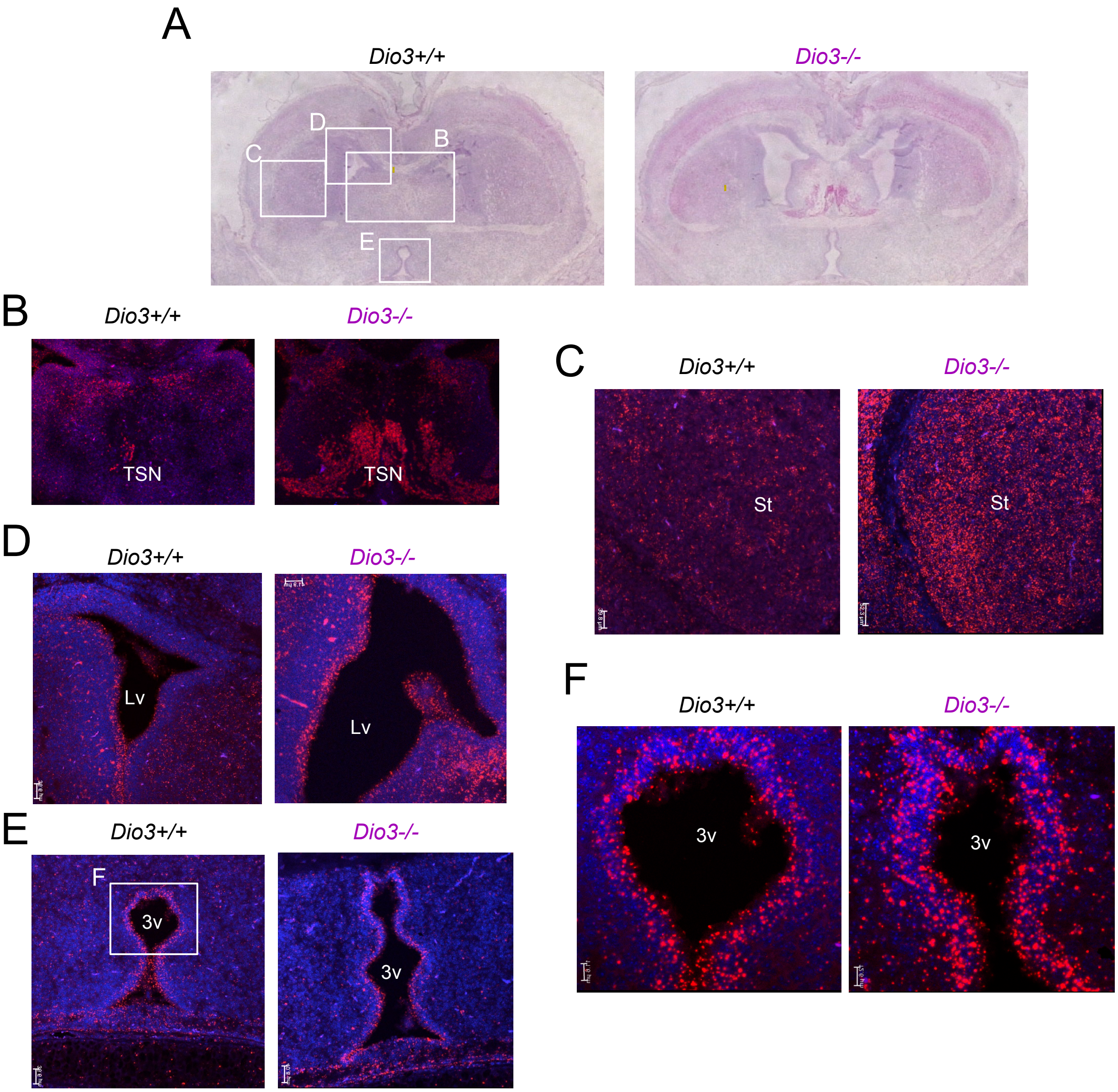

Supplementary Figure 1. *In situ* hydridization of *Klf9* mRNA in the brain of E18.5 *Dio3+/+* and *Dio3-/-* mice. Light microscope (A) and fluorececescent images (B-F) show *Klf9* mRNA expression in red. A, Full coronal section of the brain close to bregma. B, Triangular septal nucleus (TSN). C, Striatum (St). D, Lateral ventricle (Lv). E and F, Third ventricle (3v) . Images are representative of two different male mice per genotype. White rectangles and associated letters indicate the areas that are amplified in corresponding panels. Images are representative of two different male mice per genotype.

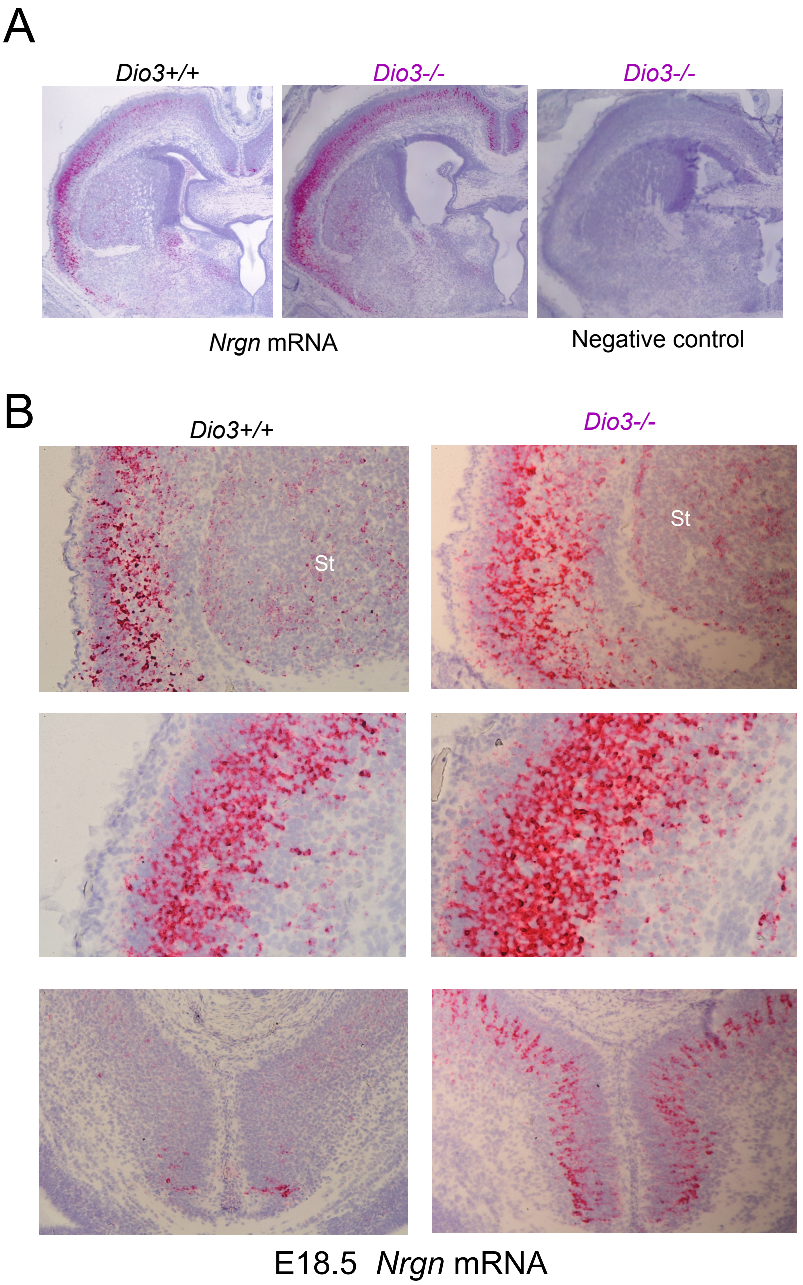

Supplementary Figure 2. In situ hydridization of *Nrgn* mRNA in the E18.5 brain of *Dio3+/+* and *Dio3-/-* mice. *Nrgn* mRNA (red) is elevated in the different cortical and striatal regions of the *Dio3-/-* brain (A), including the temporal cortex (B,Top), parietal cortex and striatum (St) (B, middle) and cingular/prefrontal cortex (B, bottom). Images are representative of two different male mice per genotype. Negative control (A) was hybridized with a bacterial probe.

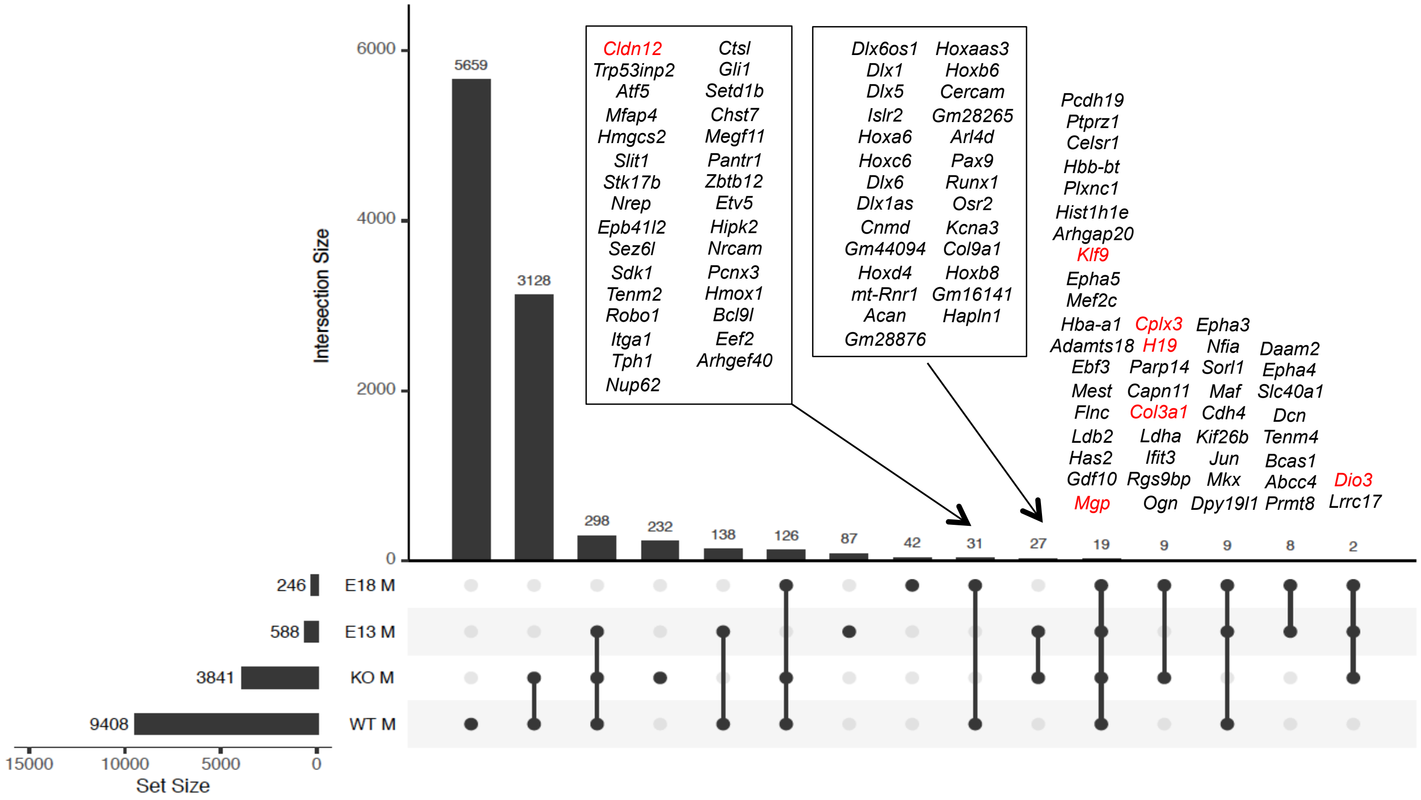

Supplementary Figure 3. Differentially expressed genes (DEGs) in the developing *Dio3+/+* and *Dio3-/-* fetal brain. Upset plot representing the overlap of four groups of DEGs: DEGs in *Dio3-/-* vs *Dio3+/+* mice at E18.5 (E18 M, 246 genes, adjusted P<0.05); DEGs in *Dio3-/-* vs *Dio3+/+ mice* at E13.5 (E13 M, 588 genes, adjusted P<0.05); DEGs in *Dio3-/-* mice at E18.5 vs E13.5 (KO M, 3841 genes, adjusted P<0.01); DEGs in *Dio3+/+* mice at E18.5 vs E13.5 (KO M, 9408 genes, adjusted P<0.01). (Genes shown in red have been validated by qPCR in the present work).
